# Supporting anticoagulant treatment decision making to optimise stroke prevention in complex patients with atrial fibrillation: a cluster randomised trial

**DOI:** 10.1186/s12875-020-01175-0

**Published:** 2020-06-08

**Authors:** Melina Gattellari, Andrew Hayen, Dominic Y. C. Leung, Nicholas A. Zwar, John M. Worthington

**Affiliations:** 1 Department of Neurology, Institute for Clinical Neurosciences, Neuroscience Research, Royal Prince Alfred Hospital, Missenden Road, Sydney Local Health District, Camperdown (Sydney), New South Wales 2050 Australia; 2 Ingham Institute for Applied Medical Research, 1 Campbell Street, Liverpool, New South Wales 2170 Australia; 3 Faculty of Health, University of Technology Sydney, 15 Broadway, Ultimo, New South Wales 2007 Australia; 4 South Western Sydney Clinical School UNSW, Liverpool, Australia; 5 Department of Cardiology, Liverpool Health Service, Sydney South West Local Health District, Clinical Services Building, Elizabeth Street, Liverpool (Sydney), New South Wales 2170 Australia; 6 Faculty of Health, Sciences and Medicine, Bond University, 14 University Drive, Robina, Queensland 4226 Australia

**Keywords:** Atrial fibrillation, Knowledge translation, Stroke prevention

## Abstract

**Background:**

Anticoagulation for preventing stroke in atrial fibrillation is under-utilised despite evidence supporting its use, resulting in avoidable death and disability. We aimed to evaluate an intervention to improve the uptake of anticoagulation.

**Methods:**

We carried out a national, cluster randomised controlled trial in the Australian primary health care setting. General practitioners received an educational session, delivered via telephone by a medical peer and provided information about their patients selected either because they were not receiving anticoagulation or for whom anticoagulation was considered challenging. General practitioners were randomised to receive feedback from a medical specialist about the cases (expert decisional support) either before or after completing a post-test audit. The primary outcome was the proportion of patients reported as receiving oral anticoagulation. A secondary outcome assessed antithrombotic treatment as appropriate against guideline recommendations.

**Results:**

One hundred and seventy-nine general practitioners participated in the trial, contributing information about 590 cases. At post-test, 152 general practitioners (84.9%) completed data collection on 497 cases (84.2%). A 4.6% **(**Adjusted Relative Risk = 1.11, 95% CI = 0.86–1.43) difference in the post-test utilization of anticoagulation between groups was not statistically significant (*p* = 0.42). Sixty-one percent of patients in both groups received appropriate antithrombotic management according to evidence-based guidelines at post-test (Adjusted Relative Risk = 1.0; 95% CI = 0.85 to 1.19) (*p* = 0.97).

**Conclusions:**

Specialist feed-back in addition to an educational session did not increase the uptake of anticoagulation in patients with AF.

**Trial registration:**

ANZCTRN12611000076976 Retrospectively registered.

## Background

Affecting 1 in 20 people over the age of 65 and one in 10 people older than 75 years [1], atrial fibrillation (AF) is associated with a five-fold increased risk of ischaemic stroke [2]. AF-related strokes are more likely to be fatal and disabling than those occurring in patients without AF [3], representing a significant burden for individuals, their families and the wider the community.

The long-standing evidence-practice gap in AF management is widely documented [4–10]. Evidence from randomised controlled trials confirms anticoagulation as the most effective antithrombotic to prevent stroke in AF [11]. However, at least one-half of people with AF do not receive anticoagulation and up to 85% experiencing an acute ischaemic stroke are either not anticoagulated or have sub-therapeutic anticoagulation [5, 7, 8].

General practitioners (GPs) have considerable reservations about prescribing anticoagulants, appearing to be *“more influenced by the events they induce (bleeds) than the events potentially prevented, in this case devastating strokes”* [12]. However, the benefits of anticoagulation outweigh the risks for the majority of people with AF, including for those with elevated bleeding risks such as the elderly [4–10]. Only around 2% of people with AF have an absolute contraindication to oral anticoagulants [10].

Barriers to evidence-based management must be overcome to minimise stroke risk. Implementation strategies such as academic detailing and audit and feedback may increase evidence-based practices [13, 14]. Opinion led educational programs may also effect change in patient management [15]. At the time of designing the study reported here, to our knowledge, there were only two randomised evaluations of implementation strategies designed to promote anticoagulant prescribing specifically targeting GPs. Ornstein et al. [16] implemented quarterly audit and written feedback reports about the management of several conditions, including AF, randomising 10 out of 20 practices to receive educational site visits and inter-practice meetings to share approaches to improve patient management. Changes in anticoagulant prescribing did not significantly differ between study arms. Audit and feedback reports presented the percentage of anticoagulated AF patients and were not focussed on individual patient cases for which tailored and specific advice may be necessary to overcome barriers to anticoagulant prescribing. Several stroke risk factors were simultaneously targeted, which may have displaced attention from managing AF to arguably less challenging care, such as blood pressure, glucose and lipid level monitoring, which were successfully modified. In another trial, a multifaceted intervention including an educational outreach visit increased AF case-finding but not appropriate antithrombotic prescribing [17]. The effect on anticoagulant prescribing was not reported. In Australia, a non-randomised study found that guideline dissemination and educational outreach targeting GPs increased warfarin use demonstrating the promise of these strategies [18].

Specialist opinions about how to best manage AF may be highly influential, guiding GP decision-making. In everyday practice there are limited opportunities for GPs to liaise with specialists for educational purposes. In one randomised controlled trial of stroke survivors, including 32 patients with AF, a model encouraging shared care between discharging specialists and GPs mediated by a nurse educator improved stroke risk management [19].

We therefore conducted a randomised controlled trial of an intervention targeting barriers to anticoagulation prescribing. We evaluated the impact of specialist communication about stroke prevention exclusively in AF, providing GPs with the opportunity to discuss specific patient cases and barriers to management with a medical peer, followed by expert decisional support to consolidate decision making. We hypothesised that a significantly higher proportion of cases managed by GPs provided with expert decisional support would receive anticoagulation compared with cases whose GPs received academic detailing in the form of an educational session alone.

## Methods

The study protocol has been published [20] and methods are summarised here. The study adheres to consort criteria for the reporting of cluster randomised controlled trials. The study was conducted between 2010 to 2012, incorporating GP recruitment commencing in 2010 and final post-test data collection in 2012.

### Setting

Almost 90% (88.6%) of the full-time equivalent Australian GP workforce is in private practice [21] and around 85% of Australian residents consult a GP at least once a year [22]. Private practice encompasses primary care assessments, preventive medicine, the ongoing care of patients with chronic illness, care of minor acute illness, hospital referrals and access to specialist and diagnostic services. Residents can consult any GP of their choosing irrespective of residential location. Consultation costs are subsidised by Australia’s universal health insurance scheme.

### Participant recruitment

As described elsewhere [20], a random sample of GPs from across Australia was selected and approached by mail. GPs participating in another study conducted by the authors were not contacted. We first undertook a pilot of recruitment methods to inform the number of GPs we needed to contact to achieve the required sample size. This was followed by two phases of recruitment (2010 and 2011), during which we carried out two trials of response-aiding strategies [20], the results of which will be reported elsewhere. GPs were asked to register their interest in the program and those so doing were mailed further information and a consent form. Trainee GPs and GPs who indicated that they were not in active private practice or whose practice did not include elderly patients were considered ineligible.

Consenting doctors received written educational materials followed by an educational session, delivered via telephone by a medically trained peer, also a GP. The educational session was consistent with academic detailing as the session was designed to be interactive, with peers detailing the mailed educational materials with the GP participants. The GP peers reinforced appropriate practice and conveyed evidence-based information to address practice that was not consistent with recommended management. We use the term “academic detailing” to label the interactive one-to-one educational component of the program and this term is used from here on.

The content of the educational materials, reported in our protocol [20], was mirrored during the academic detailing session to reinforce messages. Briefly, medical peers discussed the epidemiology of AF and stroke outcomes of AF-related stroke, risk calculation of stroke and expected risk reduction associated with anticoagulation and antiplatelet medication. Stroke risk was calculated using the CHADS_2_ score (one point each for Congestive Heart Disease, Hypertension and Age over 75 and two points allocated for prior history of stroke or transient ischaemic attack) [23] with scores ranging from 0 for patients without any CHADS_2_ risk factors to 6 for those with all CHADS_2_ comorbidities. Both medical peers and GP participants had access to a table reporting baseline stroke risk per 1000 patient years for each value of the CHADS_2_ score based on those reported by Gage, Waterman, Shannon et al. [23]. The absolute risk with either warfarin or aspirin was presented to GPs calculated by applying an estimated benefit of a 67% relative risk reduction for warfarin and a 21% relative risk reduction for aspirin [24]. For example, a CHADS_2_ risk score of 3 corresponds to an absolute annual risk of stroke of 5.9%, reduced by 67% and 21% in relative terms to 1.9% with warfarin and 4.7% with aspirin, respectively.

To explore barriers, GPs were asked how many out of 10 patients with AF over the age of 65 they would anticoagulant. Medical peers asked GPs what would characterise patients not receiving anticoagulation and then systematically addressed reasons offered against anticoagulation. Information predominately pertained to warfarin as the only government subsidised anticoagulant available for managing AF during the course of the study.

### Pre-test data collection informing primary and secondary outcomes

During the academic detailing session, GPs provided details about their selected AF cases, including age, sex, type of AF (paroxysmal versus chronic) and stroke risk factors to enable calculation of the CHADS_2_ score [23]. History and current use of antithrombotic medication were also determined. Medical history relating to actual or perceived barriers to anticoagulation were reported. We advised GPs to identify 3 to 4 AF patients during the first and at least five patients during the second phase of recruitment who were not receiving anticoagulation or for whom management was challenging. Therefore, the intervention was designed to support GP decision-making where decisions to prescribe anticoagulation was perceived to be complex or uncertain thus identifying patient cases in whom barriers to anticoagulation would be encountered. Medical peers and GPs discussed relevant guideline recommendations for either anticoagulation or antiplatelet medication based on stroke risk and the American Chest Physician Guidelines current at the time the study was conducted [25]. Safety issues were addressed including combination antithrombotic treatment, effect of hypertension on stroke risk, anticoagulant use in patients with a history of intracerebral haemorrhage and regulatory authority safety alerts for direct oral anticoagulants (DOACs), where applicable. GPs were then given the opportunity to pose questions to be answered by specialists.

### Randomization and allocation concealment

Academic detailers noted the date and time of the academic detailing session indicating entry into the study. GPs were ordered according to their entry into the study and randomised by a statistician independent to the research team to either receive expert decisional support or to academic detailing alone, receiving decisional support after the post-test phase was completed. Block randomization was stratified according to the number of cases per GP (≤ 2 or > 3), using a block size of two, four or six, with block size randomly varied.

### Expert decisional support intervention

**Fig. 1:**
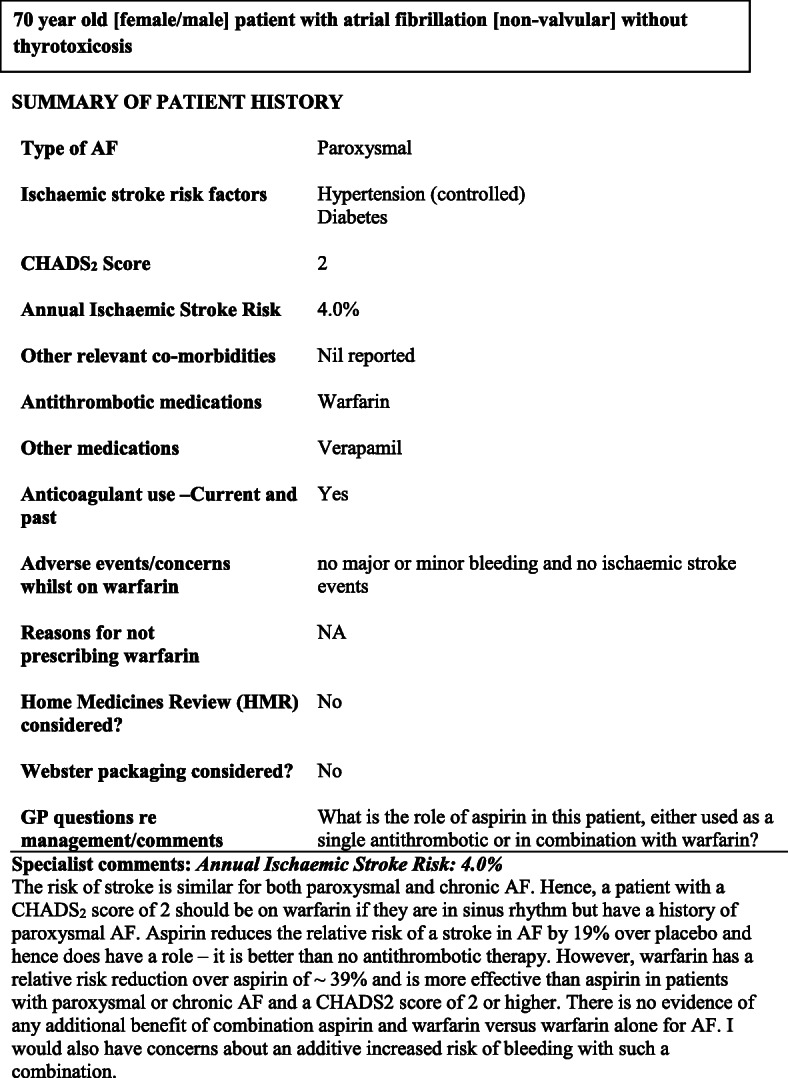
Expert summary delivered to GPs enrolled in the STOP-STROKE in AF study, 2010–2012. While this information is based on a real patient, demographic and disease characteristics have been modified to derive this hypothetical patient case. First published in Gattellari M, Worthington JM, Leung DY, Zwar. Supporting treatment decision making to optimise the prevention of stroke in atrial fibrillation: the STOP STROKE in AF study. Protocol for a cluster randomised controlled trial. Implementation Science, 2012; 7:63 10.1186/1748-5908-7-63 Creative Commons Attribution License 4.0

Information completed during academic detailing sessions was entered into an excel spreadsheet, populated into a one to two-page document and forwarded to experts by the first author. Specialists in neurology, cardiology or geriatric medicine provided written feedback, commenting on existing risk management and providing recommendations for antithrombotic treatment. A hypothetical example of summaries mailed to GPs has been published [20] and is reproduced here (Fig. 1).

### Academic detailing only group

GPs randomised to receive academic detailing only were mailed a summary of the proforma without expert specialist feedback. Proformas included a summary of the patient’s risk of ischaemic stroke and safety messages were re-stated, where relevant (also included on the summary for GPs allocated to expert decisional support).

### Post-test data collection and outcomes

Post-test assessment was scheduled within 12 weeks of the delivery of expert decisional support or patient summaries for GPs allocated to the academic detailing only arm.

The primary outcome was the proportion of cases recorded by GPs at post-test as currently receiving anticoagulant treatment, either with warfarin, clexane or dabigatran, the only DOAC first made available for managing AF in Australia from June 2011 through an industry funded familiarisation program. We selected anticoagulation as the primary outcome as barriers to the wider use of warfarin were predominately targeted given the evidence-based gap in managing AF related to concerns about anticoagulation and not antiplatelet use [7, 9, 10]. This outcome was also considered robust against reporting bias as treatment is readily ascertained by prescription records.

Secondary endpoints included antithrombotic treatment defined as appropriate if receiving 1) aspirin, 2) either an anticoagulant or antiplatelet, or 3) an anticoagulant if their baseline CHADS_2_ scores were 0, 1, or 2+, respectively, according to evidence-based guidelines current at the time of the study [25]. As an additional secondary outcome, we qualified the above definition of “appropriate” antithrombotic treatment for patients receiving warfarin if their six most recent INR results indicated at least monthly assessment and at least four were within therapeutic range (ie, within 2.0–3.0) [26, 27]. Patients younger than 65 years of age were excluded from the analysis of these secondary endpoints as pre-specified in our protocol [20] because the CHADS_2_ score was validated in patients aged over the age of 65 years.

“Appropriate” antithrombotic treatment was assessed using CHADS_2_ risk factors ascertained at baseline as the assessment and recording of these comorbidities during the course of the trial could have been influenced by the intervention itself potentially leading to differential ascertainment between groups. As we could not independently assess stroke risk factors at post-test, this outcome was designated a secondary outcome. Thus, CHADS_2_ comorbidities were recorded prior to randomisation to avoid biased ascertainment.

During the course of the study, validation of the CHA_2_DS_2_-VASc score [28] was published at which point we incorporated this information into the education program. To ensure consistency across all cases, however, we used the CHADS_2_ score for outcome ascertainment as per the original protocol.

Levels of decisional conflict about warfarin were measured using a five-item scale [29], adapted by specifying warfarin and/or its prescription within each statement. For each patient, GPs indicated their level of agreement to six statements to assess the extent to which GPs had reservations about prescribing warfarin for the patient. Summed item scores ranged from 6 to 30 with higher scores indicating a greater degree of uncertainty about whether or not to prescribe warfarin.

### Sample size estimate

We required a total of 340 patients in total to detect a 15% difference in the primary outcome, assuming a 50% baseline rate of anticoagulant prescribing [30]. This estimate was multiplied by a design effect of 1.06, accounting for the clustered data, assuming an intraclass correlation coefficient of 0.029 [27], and, to produce an anticonservative estimate for the required number of GPs, an average of three patients per GP. We inflated the total sample size of 361 patients (1.06 × 340 = 361) by 20% to adjust for losses to follow-up, producing a final estimate of 452 patient cases (226 per group) and 152 GPs (76 per group).

### Statistical analysis

Analyses were carried out by a biostatistician (co-author Andrew Hayen) blinded to group allocation. The analyses were intention-to-treat with patient data analysed according to group allocation. Log-binomial models were fitted to obtain relative risks for the analysis of dichotomous outcomes, while differences in decisional conflict scores were analysed using multiple linear regression. All analyses adjusted for the randomization stratum and accounted for clustering of patient data using generalised estimating equations. Criteria for statistical significance was a *p*-value of less than 0.05. The significance of sub-group analyses was tested using interaction terms. Analyses were performed using SAS software (SAS institute Inc., Cary, USA).

## Results

### Recruitment

**Fig. 2:**
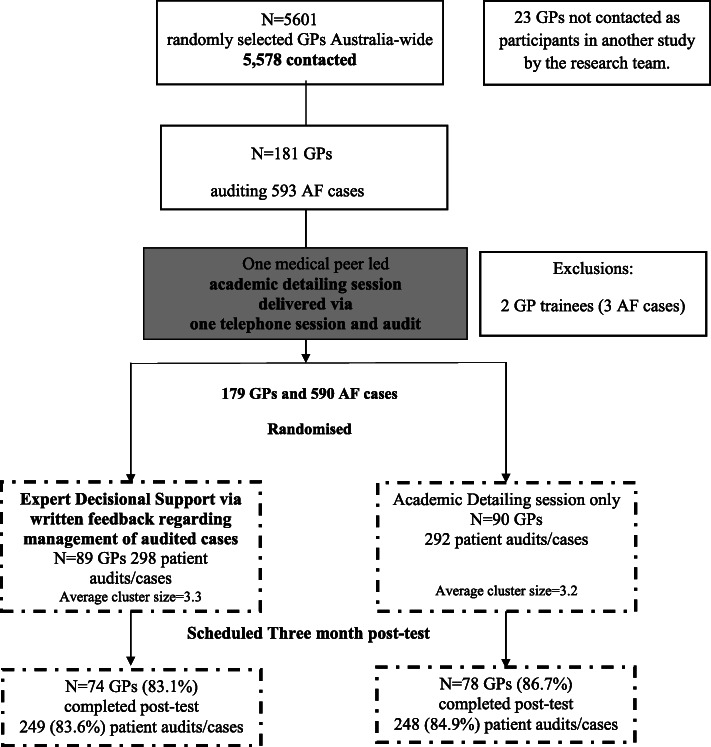
CONSORT Flow Diagram, STOP-STROKE in AF study. 2010–2012

Of 5601 randomly selected GPs located around Australia, we contacted 5578 of whom 948 (17.0%) expressed interest in receiving further information. Of these, 181 (19.1% or 3% of all GPs approached) consented and completed the academic detailing session providing baseline data for 593 patients. Two GP trainees contributing three cases were excluded prior to randomization (Fig. 2), yielding a final baseline sample of 179 GPs and 590 cases. One-hundred and 18 GPs reported being in full-time practice (65.9%, including three missing responses), which is comparable with the national GP workforce in Australia during 2010/2011 (*N* = 17,951 out of 27,076 GPs or 66.3% in “full-time service”; http://www.health.gov.au/internet/main/publishing.nsf/content/general+practice+statistics-1).

Eighty-nine GPs reporting 298 patient cases were randomly allocated to receive expert decisional support, while 90 GPs providing data on 292 cases received academic detailing only (Fig. 2). Of the 590 eligible patients, 48.0% were female (*N* = 283), while 59.0% (*N* = 350) were aged 75 years or older. Half (*N* = 293, 49.7%) were receiving anticoagulation at baseline.

Over-two thirds of patients (*N* = 401, 68.0%) had a CHADS_2_ score of 2 or more. Only 51 (8.6%) had a CHADS_2_ score of 0. Baseline patient characteristics appeared balanced between groups (Table 1). Of the 179 enrolled GP, 152 completed 497 post-test audits. The audit post-test completion rate was 84.2% and did not significantly differ between groups (χ^2^_(1)_ = 0.47, *p* = 0.49) (Fig. 2).

**Table 1:** Baseline comparison of patient characteristics by group, Stop-Stroke in AF study 2010–2012

Variable	Academic Detailing+ Expert Decisional Support n (%) ***N*** = 298	Academic Detailing n (%) ***N*** = 292	Total n (%) ***N*** = 590
**Sex**			
Male	152 (51.0)	155 (53.1)	307 (52.0)
Female	146 (49.0)	137 (46.9)	283 (48.0)
**Age Group**			
< 65	27 (9.1)	30 (10.3)	57 (9.7)
65–74	94 (31.5)	89 (30.5)	183 (31.0)
75–84	117 (39.3)	114 (39.0)	231 (39.2)
85+	60 (20.1)	59 (20.2)	119 (20.2)
Mean (SD)	76.5 (9.5)	76.5 (9.2)	76.5 (9.4)
Median (IQR)s	77.5 (70–84)	77.0 (70–83)	77 (70–84)
**CHADS**_**2**_**score**			
0	22 (7.4)	29 (9.9)	51 (8.6)
1	57 (19.1)	71 (24.3)	128 (21.7)
2+	212 (71.1)	189 (64.7)	401 (68.0)
N/A (Cases with valvular AF)	7 (2.3)	3 (1.0)	10 (1.7)
Mean (SD)	2.4 (1.4)	2.2 (1.4)	2.3 (1.4)
Median (IQR)	2 (1–3)	2 (1–3)	2 (1–3)
**Baseline anticoagulation**			
Yes	153 (51.3)	139 (47.6)	292 (49.5)
Previous Use	46 (15.4)	47 (16.1)	93 (15.8)
No	96 (32.2)	105 (36.0)	201 (34.1)
Missing	3 (1.0)	1 (0.3)	4 (0.7)
**Type of antithrombotic**			
Aspirin	92 (30.9)	102 (34.9)	194 (32.9)
Clopidogrel	12 (4.0)	13 (4.5)	25 (4.2)
Combined Antiplatelet^a^	12 (4.0)	17 (5.8)	29 (4.9)
Anticoagulant+Antiplatelet^b^	13 (4.4)	17 (5.8)	30 (5.1)
Warfarin	125 (41.9)	104 (35.6)	229 (38.8)
Dabigatran	14 (4.7)	16 (5.5)	30 (5.1)
Clexane	1 (0.3)	2 (0.7)	3 (0.5)
None	29 (9.7)	21 (7.2)	50 (8.5)

*N/A* Not applicable

*SD* Standard Deviation

*IQR* Interquartile Range

^a^Includes aspirin plus dipyridamole

^b^Four patients were using dabigatran

There was evidence that cases lost to follow-up had a higher stroke risk and were more likely to be receiving anticoagulation at baseline. Of the 93 cases lost to follow-up (15.8%), 58.1% (*N* = 54) received anticoagulation at baseline, compared with 47.5% (*N* = 236/497) of those with follow-up data, a near significant difference (χ^2^_(1)_ = 3.51, *p* = 0.06). Mean CHADS_2_ scores were lower in patients lost-to-follow-up (Mean = 2.1, SD = 1.3), compared with those for whom follow-up data were obtained (Mean = 2.3, SD = 1.4), although differences were not statistically significant (t_578_ = 1.75, *p* = 0.08). There were no significant differences in sex, age group, median or mean age or median CHADS_2_ scores or CHADS_2_ categories between patients for whom post-audit data were and were not collected (p’s > 0.10).

### Primary outcome

GPs receiving expert decisional support reported that 52.2% (*N* = 130) of their patients were anticoagulated at post-test. By comparison, 47.6% (*N* = 118) of patents were anticoagulated at post-test by GPs receiving academic detailing alone. This difference was not statistically significant (Adjusted Relative Risk = 1.11, 95% CI = 0.86 to 1.43) (*p* = 0.42) (Table 2).

**Table 2:** Comparison of primary and secondary outcomes reported at post-test, by group, Stop Stroke in AF study 2010–2012

Variable	Expert Decisional Support n/N (%)	Academic Detailing alone n/N (%)	Effect Estimate	95% CI	***p***-value
***Primary outcome***^a^					
Receiving anticoagulant therapy (*N* = 497)	130/249 (52.2)	118/248 (47.6)	RR = 1.11	0.86–1.43	0.42
***Secondary outcomes***					
Appropriate antithrombotic treatment (*N* = 443) ^b^	136/223 (61.0)	134/220 (60.9)	RR = 1.00	0.85–1.19	0.97
Appropriate antithrombotic incorporating quality control criteria (*N* = 443) ^b^	91/223 (40.8)	99/220 (45.0)	RR = 0.91	0.73–1.14	0.43
Decisional Conflict (*N* = 497)	Mean = 16 95% CI = 15.3–16.7	Mean = 16.6 95% CI = 16.0–17.2	β = −0.50	−1.79-0.77	0.44

Academic Detailing = Referent group

^a^Intraclass correlation coefficient for the primary outcome was 0.35

^b^Outcome was assessed only for patients aged 65 years or older as specified in protocol

### Secondary outcomes

Sixty-one percent of patients in both groups received appropriate antithrombotic management at post-test according to evidence-based guidelines (Adjusted RR = 1.0, 95% CI = 0.84 to 1.18) (*p* = 0.97). Similarly, there was no difference between groups in the proportion of patients receiving appropriate antithrombotic management incorporating quality INR control criteria (40.8% versus 45.0%; Adjusted RR = 0.91, 95% CI = 0.73–1.14, *p* = 0.43). Planned sensitivity analyses for these secondary outcomes including patients younger than 65 years of age were not carried out given the null findings of the main analyses. Mean levels of decisional conflict were equivalent between groups (*p* = 0.44) (Table 2).

**Fig. 3:**
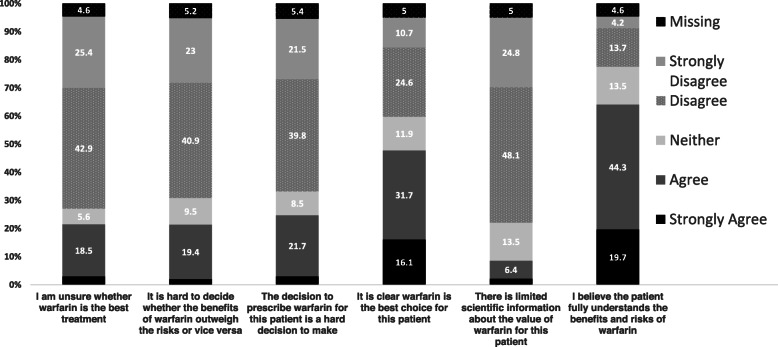
Decisional conflict item scale responses (%), *N* = 497. The Stop Stroke in AF Study, 2010–2012

Responses to individual items comprising the decisional conflict scale are shown in Fig. 3. For around one-in-five cases, GPs “strongly agreed” or “agreed” that they were unsure warfarin was the best treatment for the patient and whether the benefits of warfarin outweighed the risks or vice-versa. For one-third of selected cases, GPs “disagreed” or “strongly disagreed” that it was clear warfarin was the best treatment for the patient.

### Subgroup analyses

Pre-specified subgroup analyses tested differences between groups on the primary outcome according to baseline CHADS_2_ scores (0, 1, or ≥ 2), anticoagulation use at baseline (yes versus no) and patient sex and age (< 65 years, 65–74 years, 75–84 years, ≥85 years). There was no evidence that the impact of group allocation on the primary outcome depended on patient age, sex, baseline CHADS_2_ score or baseline use of anticoagulation (p-values_interaction_ > 0.05).

Amongst patients with available follow-up data, 214 out of 236 (90.7%) receiving anticoagulation at baseline were still receiving it at post-test, while 11 out of 85 whose GPs reported at baseline their anticoagulation medication had been discontinued resumed treatment at post-test (12.9%). Of the 176 patients with no history of anticoagulation use at baseline, 23 (13.1%) received it at post-test.

## Discussion

### Main findings

Specialist feed-back in addition to a self-audit facilitated by peer-led academic detailing did not increase anticoagulant prescribing in Australian general practice. We addressed GPs’ concerns about specific cases and detailed medical histories informed specialist feedback. This is in contrast to traditional methods for providing feedback using aggregated statistics and benchmark targets. Yet this novel method was not effective in promoting anticoagulant prescribing.

### Comparison with other studies

Audit and feedback and academic detailing do not consistently promote evidence-based practice [13, 14] and our findings are in step with other evaluations of these strategies undertaken in a variety of contexts. Results are also consistent with findings from another study evaluating a multifaceted intervention for anticoagulation prescribing in AF, incorporating academic detailing and audit with feedback as we have done [16]. Moreover, three secondary stroke prevention trials did not report increased rates of anticoagulant prescribing for patients in AF in response to structured discharge care plans or nurse-led care [19, 31, 32].

Frequent feedback, using more than one delivery format, implementing action plans and providing targets enhance the effectiveness of audit and feedback [14]. While feedback here was delivered twice, verbally and in writing, we did not include action plans or specific targets and this may have underpinned the null findings. While feedback delivered by stroke specialists was hypothesised to be persuasive, the influence of experts may have been limited because they were not otherwise known to GPs nor involved in the care of their patients. This finding is consistent with results from another community-based study evaluating written, evidence-based summaries provided by opinion leaders, showing no improvement in the management of heart failure or ischaemic heart disease [33].

The intervention focussed on GPs while patients were not engaged. A recent study reported a small (3%) but significant increase in anticoagulant prescribing attributable to a computerised decision support tool activated during interactions between physicians and patients [34], consistent with results from an earlier study employing a computerised risk assessment tool to guide real-time decision making [35]. In contrast, another recent study found no effect of an intervention using email notifications reporting patient-specific stroke risk and guideline recommendations for antithrombotic treatment [36]. Evidence from other randomised and non-randomised trials suggests simply having access to support tools, such as risk calculators, electronically generated feedback reports and automated prompts in anticipation of patient visits does not impact prescribing rates [37–39]. Taken together, findings from this and our own research imply that interventions delivered outside the context of the consultation are ineffective, yet providing opportunities for GPs to engage patients in discussions during real-time decision making may drive changes in prescribing behaviour.

The selection of patients by GPs in whom anticoagulation has been challenging may have made change especially difficult to achieve as there was no meaningful differences in anticoagulation rates from baseline to post-test in either group. The intervention therefore can be considered ineffective for patients selected in this way and the results may not generalise to all AF patients in general practice, for example, patients newly diagnosed with AF who are naïve to anticoagulants or those with less challenging needs. The impact of expert decisional support did not differ according to baseline patient characteristics, including baseline stroke risk. Therefore, there was no evidence that the impact of the intervention would have differed in patients with varying stroke risk. We acknowledge, however, that complex needs not reflected in the CHADS_2_ score, such as a patient’s social and psychological circumstances, their cognitive capacity and adherence to medication may work against efforts to promote anticoagulation. While we did not systematically assess such barriers, these factors may have been relevant for the GPs. Moreover, there was no change in either group from baseline to post-test.

### Implications for current practice

Our study was carried out as guidelines were evolving away from use of the CHADS_2_ score to the CHA_2_DS_2_-vasc score for risk stratification although US guidelines continued to recommend the use of CHADS_2_ sore in 2012 [40]. We had incorporated the CHA_2_DS_2_-vasc scheme to ensure GPs were aware of impending changes to local guidelines and feedback from experts also utilised this scheme where relevant. According to current recommendations [41], more AF patients are identified as suitable for anticoagulation, including those younger than 65 years of age. In this study, around 90% of patients were eligible for anticoagulation using the CHADS_2_ risk stratification scheme and therefore would have also been eligible under the CHA_2_DS_2_-vasc scheme. The main impact of the change in guidelines would have related to the 57 patients under 65 years of age in our study.

Antiplatelet medication has been largely superseded for the management of AF and recommendations now advise anticoagulation is to be preferred even in relatively low risk patients [42]. Real-world data indicates that a significant proportion (~ 15%) of patients with AF are prescribed antiplatelets, [43] and a recent study demonstrates no change in the proportion of patients prescribed aspirin since the change in guidelines [44], highlighting that current day treatment for AF remains sub-optimal for a sizeable proportion of patients.

The study was carried out before DOACs became widely available and at a time when their subsidised release under Australia’s universal medical insurance scheme was imminent. Reluctance to prescribe warfarin may be intransigent. DOACs are perceived to be more convenient [45] with a proven lower risk of intracerebral haemorrhage [11] and these characteristics may leverage wider uptake of anticoagulation. Expert decisional support may have greater traction in the current post-DOAC era as DOACs may be an “easier sell” if GPs are more pre-disposed to using these drugs over warfarin. However, our results are consistent with several randomised and non-randomised evaluations of implementation strategies for reducing stroke risk in AF which mainly report null findings or small differences in the uptake of anticoagulants in the pre and post-DOAC era [32, 36–39]. Taken together, these findings indicate that shifting prescribing practice remains challenging.

The recently published multicentre, multi-national IMPACT-AF study conducted in low to middle-income countries, incorporated both patient and GP education and closely monitored individual patients over a 12-month period [46]. The study reported a 12% increase in anticoagulant prescribing over one-year compared with a 3% change in the usual care control arm. The results of IMPACT-AF may owe its success to persistent and sustained contact with both GPs and patients and the availability of DOACs. However, almost 80% of patients on anticoagulants were receiving warfarin at post-test, indicating a continued role for warfarin.

The study findings can be applied to current practice where warfarin is considered and continues to be used and, more generally, if the barriers to warfarin use also impede the uptake of DOACs. In Australia and elsewhere, DOAC dispensing has increased concomitantly with decreasing warfarin, although warfarin remains commonly prescribed [44, 47–55], and recent surveys show that warfarin is favoured amongst a significant proportion of clinicians [56], particularly for patients in whom use is established. In Australia, reports indicate that around one-quarter to one-third of initial anticoagulant prescribing is for warfarin [48, 49, 54]. There is also growing evidence that DOAC prescribing behaviour is influenced by the same perceived barriers that limited the wider use of warfarin indicating that DOACs do not appear to have bridged the evidence-practice gap as initially hoped and are subject to the same perceived limitations of warfarin. Recent studies report a high proportion of uncoagulated patients, for example, 32% [43] and 57% [52], and evidence also demonstrates that doctors remain cautious about bleeding risks, leading to non-evidence-based DOAC use. One large multicentre study of general practice patients showed that 40% of patients with AF and 40% of those at a high risk of stroke were not receiving anticoagulation despite trends towards increasing DOAC use [51]. Moreover, DOACs have been preferentially prescribed to younger patients, those with lower bleeding risks, fewer comorbidities and/or with lower stroke risk [50–53]. This has also been shown to occur in Australia, where patients with lower comorbidity burden are preferentially prescribed higher dose DOACs [57]. The underuse of anticoagulation in patients over the age of 85 remains significant [43]. In one analysis of over 100,000 anticoagulated AF patients, DOACs were associated with a higher risk of ischaemic stroke and shorter prothrombin time compared with warfarin use [58], suggesting under-coagulation in patients using these newer agents. Under-dosing is common [47, 59–62] and has been associated with an increased risk of thromboembolic events and mortality, although findings have not been consistent [47, 59–62]. Contraindicated use or use inconsistent with recommendations is another concern [60, 61]. In an Australian study of AF patients admitted to hospital, 40% were judged to have been prescribed a DOAC inappropriately. Specifically, DOACs were contraindicated in around one-in-eight patients, with both under-dosing and overdosing reported in patients (~ 15 and 10%, respectively) [61].

Moreover, discontinuation of all oral anticoagulants is prevalent. In a study analysing data from over 45,000 newly-anticoagulated patients in the United States, 50% receiving Apixaban, 65% receiving Dabigatran, 57% receiving Rivaroxaban and 71% receiving warfarin discontinued treatment within 1 year of initiating it and no more than 10% of patients switched to another agent [63]. In another large-scale study of over 64,000 patients, only 47% of those taking DOACs adhered to treatment for more than 80% of the time at a median of 1.1 years of follow-up, compared with 40% of those taking warfarin [64] while in another report based on over 62,000 patients newly diagnosed with AF, discontinuation of DOACs at 3 years was 46% [51]. These results underscore that stroke risk reduction in AF remans ripe for knowledge translational activity and, contrary to expectations, barriers to wider and appropriate anticoagulation persist in contemporary practice.

DOACs were heralded to bridge the evidence-practice gap in AF management. Wider use of DOACs will increase the absolute numbers of patients who may experience a bleeding event if such patients would not have previously been anticoagulated given their risk of an adverse event would have been lower without any anticoagulation. If GPs encounter more patients experiencing adverse events, this may negatively reinforce their prescribing, resulting in more cautious future use and less evidence-based prescribing. These altering perceived risks may potentially impact on prescribing intentions, motivating GPs to reduce bleeding risk (or an act of commission) at the expense of reducing stroke risk (an act of omission) [12]. For these reasons, our results may inform future studies evaluating strategies to increase anticoagulation uptake.

### Strengths and limitations

The large number of GPs involved in this study is a strength. Limitations include the unblinded self-reported outcomes and the 15% attrition rate of participants. Losses to follow-up were non-differential between groups and any bias would have likely favoured the intervention. Further, the three-month scheduled time between delivery of the intervention and post-test assessment may have limited the opportunities for GPs to change prescribing practices as recalling patients for a consultation was not specifically encouraged. Specialists to whom GPs referred patients may have been predominately responsible for determining treatment and GPs may have been reluctant to deviate from and contradict those decisions. The “control group” of the study was an active arm. Consequently, we do not know whether either expert decisional support, academic detailing or self-audit minimised discontinuation of anticoagulation compared to usual care. We do not have insights into GPs’ views about the intervention as we did not conduct qualitative interviews at the end of the study. Specifically, it would be of interest to understand the reasons GPs did not change prescribing practice despite expert recommendations. We did not ask GPs whether they or another doctor had initiated treatment and do not know to what extent this contributed to the null findings. These issues are an important focus for future research.

GPs were advised to select patients for whom management presented challenges. This made case selection less costly in comparison to an independent audit. Only half of selected patient cases were receiving anticoagulation, even though 90% had a risk of stroke justifying anticoagulant use, characteristics which are consistent with those reported by others [4, 7, 8]. We saw evidence of decisional conflict with one-in-five GPs indicating difficulties in weighing up the benefits and risks of anticoagulation with one-third disagreeing that warfarin was the best choice for patients. These findings indicate that GPs selected appropriate cases for this knowledge translation exercise and self-audit may be relied upon in future research.

The selection of patients for whom management was considered challenging may have skewed the cohort towards a group of patients with more complex needs. More than two-thirds of patients had stroke risk warranting anticoagulation and 70% had a stroke risk assessed as “high” according to their CHADS_2_ score (that is, a score of two or more); fewer than 10% were deemed at low risk of stroke. The distribution of stroke risk is skewed towards higher risk compared with registrants in multicentre registries [65], although it is comparable to another knowledge translation Australian trial of AF patients recruited from general practice [35]. An audit of 82 Australian general practices carried out at the time this study was conducted reports just over one-third of patients having high risk of stroke compared with 70% here, although, like here, fewer than 10% were considered low risk [66].

The study cohort is best described as one of motivated volunteers as only 3% of GPs initially contacted eventually participated in the study. In the context of a randomised evaluation, however, comparisons should be internally valid. The national scope of the research and our limited resources precluded more resource intensive recruitment strategies, such as those involving follow-up phone calls, site visits and repeated mailings which would have likely increased participation. The characteristics of the selected cases are in keeping with previous studies [4, 7, 8] and the proportion of GPs in full-time practice was consistent with national GP workforce statistics. Low participation rates amongst GPs in research studies is not an isolated experience. In an implementation trial carried out in the United Kingdom aimed at improving anticoagulant prescribing in AF, Holt et al. [37] recruited 8% of 570 general practices invited to participate via a mailed letter. Two Australian cluster randomised trials carried out in general practice have reported a 6% and an 8% participation rate amongst GPs located within the researchers’ local areas which were predominately urban [67, 68]. We utilised a study base of a random sample of GPs which could be expected to result in a lower participation rate compared with enrolling participants from a registry of self-nominated GPs interested in research.

To address the challenges in recruiting GPs, we deliberately approached a large number of potential participants and carried out two “Studies Within a Trial” (SWATs) evaluating various response-aiding strategies [20] at the time of initial contact. Interest in carrying out SWATs is gaining momentum to generate high quality evidence to improve trial administrative processes [69] and our findings will generate evidence to inform improved recruitment in primary health care implementation research.

## Conclusions

Expert decisional support, delivered in writing, in addition to one academic detailing session administered via telephone by a medical peer compared with academic detailing alone, did not increase anticoagulant prescribing for patients GPs identified as not receiving anticoagulation or for whom management was perceived as challenging. Tested in this way, we found no support for its usefulness where warfarin was the predominately available anticoagulant. There may be merit in testing the effectiveness of specialist support using different methods of delivery (for example, a more interactive format), more frequent contact, focussing on newly diagnosed patients naïve to anticoagulation or in the context of increasing anticoagulant use [70], in which evidence-practice gaps persist [47, 50–53, 59–62, 71]. On the basis of results reported here, expert decisional support cannot be recommended to improve anticoagulant prescribing in patients for whom anticoagulation is challenging.

## Data Availability

Data sharing was not a requirement at the time the study was conducted. Therefore, consent was not obtained from participants to share individual level data containing health information beyond the research team.

## Abbreviations

AF: Atrial Fibrillation
ANZCTRN: Australian and New Zealand Clinical Trials Registration Number
DOAC: Direct Oral Anticoagulant
GP: General Practitioner
